# Metabolic Vulnerabilities and Epigenetic Dysregulation in Myeloproliferative Neoplasms

**DOI:** 10.3389/fimmu.2020.604142

**Published:** 2020-11-30

**Authors:** Vasundhara Sharma, Kenneth L. Wright, Pearlie K. Epling-Burnette, Gary W. Reuther

**Affiliations:** ^1^Department of Leukemia, Princess Margaret Cancer Center—University Health Network, Toronto, ON, Canada; ^2^Department of Immunology, Moffitt Cancer Center, Tampa, FL, United States; ^3^Ibis Therapeutics, Tampa, FL, United States; ^4^Department of Molecular Oncology, Moffitt Cancer Center, Tampa, FL, United States

**Keywords:** myeloproliferative neoplasm, metabolism, epigenetic, EZHZ2, HDAC11, JAK2, therapy, stem cell

## Abstract

The Janus kinase 2 (JAK2)-driven myeloproliferative neoplasms (MPNs) are associated with clonal myelopoiesis, elevated risk of death due to thrombotic complications, and transformation to acute myeloid leukemia (AML). JAK2 inhibitors improve the quality of life for MPN patients, but these approved therapeutics do not readily reduce the natural course of disease or antagonize the neoplastic clone. An understanding of the molecular and cellular changes requisite for MPN development and progression are needed to develop improved therapies. Recently, murine MPN models were demonstrated to exhibit metabolic vulnerabilities due to a high dependence on glucose. Neoplastic hematopoietic progenitor cells in these mice express elevated levels of glycolytic enzymes and exhibit enhanced levels of glycolysis and oxidative phosphorylation, and the disease phenotype of these MPN model mice is antagonized by glycolytic inhibition. While all MPN-driving mutations lead to aberrant JAK2 activation, these mutations often co-exist with mutations in genes that encode epigenetic regulators, including loss of function mutations known to enhance MPN progression. In this perspective we discuss how altered activity of epigenetic regulators (e.g., methylation and acetylation) in MPN-driving stem and progenitor cells may alter cellular metabolism and contribute to the MPN phenotype and progression of disease. Specific metabolic changes associated with epigenetic deregulation may identify patient populations that exhibit specific metabolic vulnerabilities that are absent in normal hematopoietic cells, and thus provide a potential basis for the development of more effective personalized therapeutic approaches.

## Introduction

Myeloproliferative neoplasms (MPN) are BCR-ABL-negative hematological malignancies where mutations in hematopoietic stem cells (HSCs) give rise to aberrant production of myeloid clones leading to three distinct clinical phenotypes. Polycythemia vera (PV) is characterized by trilineage myeloproliferation and extramedullary hematopoiesis, notably leading to elevated red blood cells and hematocrit, while essential thrombocythemia (ET) is characterized in part by dysregulated megakaryopoiesis. In addition to aberrant myeloid cell production, myelofibrosis (MF) is characterized by reactive fibrosis in the bone marrow (1, 2). MPN patients show various symptoms including spleen and liver enlargement, fatigue, pruritus, fever, night sweats, and bone pain. PV and ET can progress to MF and MF patients are at an increased risk of developing acute myeloid leukemia (AML) and have the poorest survival (2, 3). For this reason, clinical assessment of treatment paradigms in MPN have largely been focused on MF patients (4, 5). Advances in our understanding of MPNs from both clinical and pre-clinical studies have defined cell signaling driving mutations which can co-occur with mutations in other genes, including genes that encode epigenetic regulators, that correlate with differential severity of disease (6). Diverse outcomes and responses to current targeted therapies suggest that multiple factors play prominent, yet undefined, roles in MPN pathology, and thus a better understanding of aberrant cellular processes that contribute to MPN remains needed in order to develop effective therapies. Recent preclinical work has suggested significant alterations in metabolic processes may contribute to the development and progression of MPN (7). Herein we highlight and speculate how MPN driving mutations and epigenetic regulators may contribute to reprogrammed metabolism that may provide potential liabilities that can be exploited by future therapeutic intervention for MPN.

## MPN Driving Mutations, Cell Signaling, and Targeted Therapies

Somatic driver mutations in three genes account for about 90% of all MPN cases. These include mutually exclusive activating mutations in the genes that encode Janus Kinase 2 (*JAK2*) and the thrombopoietin receptor (encoded by the myeloproliferative leukemia virus gene, *MPL*), as well as inactivating/neo-functional mutations in the gene that encodes the ER chaperone protein calreticulin (*CALR*) (6, 8). A gain of function mutation encoding JAK2-V617F is the most commonly occurring single mutation, present in more than 95% of patients with PV and 50% to 60% of patients with ET and PMF. The frequency of frameshift mutations in CALR is about 30% in ET and 25% in PMF. Activating mutations in *MPL* are present in ∼ 2% to 4% of ET patients and ~ 3% to 5% of PMF patients (6, 8). These mutations are designated drivers of human MPN in part because they induce MPN phenotypes in mouse models (9–11). As a common signaling event induced by MPN driving mutations, JAK2 activation is the critical signaling node in MPN, with MPL playing a requisite role in driving myeloproliferation driven by mutant JAK2 and CALR (12–18).

Deregulated JAK2 signaling in MPNs leads to cytokine hyper-sensitivity and enhanced activity of downstream signaling effectors such as the STAT transcription factors, as well as the ERK/MAPK and the PI3K/AKT pathways which play roles in regulating cell survival, proliferation, and apoptosis (1). Genetic removal of STAT5 impedes the MPN phenotype in MPN mouse models, and small molecule inhibitors of the ERK/MAPK pathway and the PI3K/AKT/mTOR pathway antagonize disease, further defining JAK2-mediated signals as playing important roles in MPN (19–27).

Among the current therapies, only allogenic stem cell transplantation is curative, capable of resolving bone marrow fibrosis and the malignant clone (28). Interferon-alpha is similarly promising, and while initially associated with treatment-limiting toxicities, the development of pegylated-interferon has made cytogenetic remission with this agent a real possibility (29, 30). The JAK1/2 kinase inhibitor ruxolitinib was approved by the United States Food and Drug Administration in 2011 for high risk MF patients and in 2014 for certain PV patients (31–33). Ruxolitinib provides symptomatic relief and can improve survival but generally fails to resolve the malignant clone (33–42). Fedratinib, a JAK2 and FLT3 inhibitor approved in 2019 for higher risk MF patients has similar quality of life improvement benefits as seen with other JAK2 inhibitors, but importantly can be effective in some patients who failed ruxolitinib (43–46). Time will determine the extent to which fedratinib improves critical parameters of MPN and survival. However, results from clinical experience suggest targeting JAK2 may not be sufficient or even the best option to reverse the course of disease in MPN patients, and a plethora of pre-clinical studies have suggested a potential benefit of therapeutic combinations. Many of these studies have focused on combining JAK2 inhibitors with inhibitors of signaling proteins associated with JAK2 activation with the hope of enhancing the efficacy of JAK2 inhibitor mono-therapy, which could provide therapeutic opportunities for additional patients, as well as potentially overcome JAK2 inhibitor resistance. Several of these combinations are being assessed in clinical trials (5).

## Altered Epigenetic Regulation in MPN—Opportunities for Therapeutic Targeting?

Commonly co-occurring mutations with MPN drivers are in genes that encode epigenetic regulators (6, 8). While such mutations have contributed to refining MPN patient prognostication, studies have also highlighted the potential of epigenetic regulators, or the pathways they affect, as potential therapeutic targets in MPN (6, 8, 47–51).

Some of these epigenetic regulators include DNMT3A, TET2, EZH2, ASXL1, and IDH1/2 (via effects on TET2-mediated methylation). In some cases, concomitant mutation of these genes with MPN driving mutations can enhance disease phenotypes in MPN mouse models (47, 48, 52–57). These studies suggest mutations, most often loss of function, of these epigenetic regulators may contribute to disease progression in MPN patients. In fact, the prognosis and patient response to ruxolitinib is negatively impacted by the presence of many of these mutations, with worse prognosis associated with a greater numbers of mutations present (58–62).

However, other epigenetic regulators have emerged as possible therapeutic targets in MPN. For example, targeting the activity of LSD1 in mouse models of MPN antagonizes disease development (49). The LSD1 inhibitor IMG-7289 is currently being assessed in myelofibrosis patients. Co-expression of mutant IDH1 or IDH2 with JAK2-V617F enhances MPN progression in mice, and a small molecule IDH inhibitor along with ruxolitinib provides enhanced antagonism of disease (48). Inhibitors of BET proteins, which bind acetylated histones to promote gene expression, are effective in MPN models and cooperate with ruxolitinib by antagonizing pro-inflammatory gene signatures, and such inhibitors are under clinical assessment in MF patients (63–66). The loss of EZH2 promotes the progression of MPN in mouse models and confers enhanced sensitivity of disease to BET inhibition (47). Finally, we have recently shown a role for HDAC11 in MPN, but not normal, hematopoiesis, in one of the first reports that shows isoform selective HDAC targeting specifically impedes malignant hematopoiesis without affecting the steady state and transplantation reconstitution of normal bone marrow cells. This suggests HDAC11 may be a therapeutic target to antagonize MPN with minimal adverse effect on normal hematopoiesis (50).

## MPN Drivers Deregulate Metabolic Processes

Understanding specific disease-driving effects of aberrant JAK2 signaling in hematopoietic stem and progenitor cells (HSPCs) may provide critical information regarding targets for novel therapies. Inhibition of JAK2 signaling in MPN mouse models elicits a reduced phenotypic disease burden, but it does not readily eradicate the MPN-driving and MPN-initiating cells, reminiscent of JAK2 inhibitor effects in human MPN (67–69). This suggests the HSPC signaling that drives MPN pathogenesis is resistant to the effects of direct JAK2 inhibition and that further understanding the molecular and cellular forces that drive disease are needed to identify potential vulnerabilities that can be targeted to effectively antagonize disease-driving cells. However, in addition to intrinsic abnormalities in MPN-driving cells, microenvironmental contributions play a significant role to malignant hematopoiesis in MPN, including critical roles of megakaryocytes in shaping a disease-driving hematopoietic compartment (16, 63, 70–76). Thus, understanding both intrinsic and extrinsic contributions to MPN-driving HSPCs may be critical for the development of a therapy that effectively induces remission from the neoplastic MPN clone.

Disease-driving HSPCs reside in bone marrow niche microenvironments that become remodeled during malignancy and aging (77). Characteristic of leukemic stem cell survival and expansion are metabolic changes that lead to altered sources of energy production (77, 78). For example, AML leukemia stem cells, unlike leukemic blast cells, depend on oxidative phosphorylation driven by amino acid metabolism, creating a significant therapeutic vulnerability for this disease-driving cell populations (79, 80). Interestingly, this dependency was lost in leukemia stem cells from relapsed patients, where fatty acid metabolism provided the requisite energy source, demonstrating that metabolic changes may drive malignancy and relapse (80). Perhaps more relevant to chronic phase MPN is the report of HSPC subsets that exhibit increased glycolysis associated with myeloid skewing in older people, and the possibility that this may correlate with the natural clonal hematopoiesis associated with aging (81, 82). In addition to JAK2 mutations in *DNMT3A*, *TET2*, and *ASXL1* are amongst the most commonly detected mutations associated with aging-dependent clonal hematopoiesis in populations not exhibiting hematologic malignancy (83–86). The role of metabolic reprogramming in MPN stem and progenitor cells is not well understood, although several studies have provided important observations.

The JAK2-V617F MPN-driving kinase was shown to enhance the expression the glycolytic enzyme 6-phosphofructo-2-kinase/fructose-2,6-bisphosphatase 3 (PFKFB3) (87). PFKFB3 regulates 6-phosphofructo-1-kinase activity which is a rate limiting enzyme of glycolysis (88). JAK2-V617F-expressing cells exhibited enhanced glucose uptake which correlated with elevated levels of the glucose transporter Glut1, both of which were shown to be under the control of the activity of JAK2-V617F (87). This study demonstrated that JAK2-V617F-dependent regulation of glucose uptake and PFKFB3 have the potential to contribute to enhanced lactate production and metabolic activity. Importantly, significantly elevated PFKFB3 mRNA levels were detected in PV patients compared to healthy controls. The authors demonstrated that PFKB3 expression and activity were required for optimal growth of JAK2-V617F-expressing cells *in vitro* and as tumors in mice (87). Another study evaluated the previously known increased dependence of cancer cells on free amino acids such as glutamine (89, 90). This study indicated glutaminase (GLS), an enzyme that converts glutamine into glutamate, was not found to be upregulated in primary MPN cells but provides some evidence that targeting its activity may enhance the effect of JAK2 inhibition (91). While these studies provide initial insight into the potential metabolic enzymes may provide a target to improve MPN therapies, they were mostly limited to cellular models.

**Figure 1:**
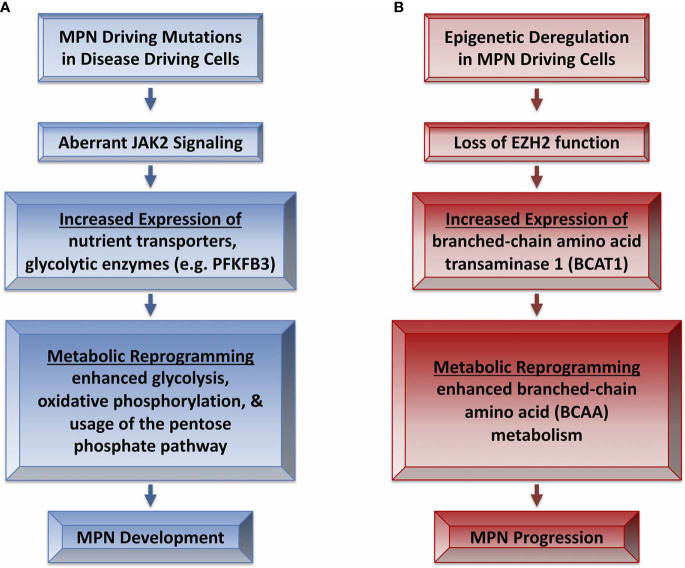
Metabolic reprogramming in MPN development and progression. **(A)** MPN driving mutations lead to aberrant JAK2 signaling that leads to metabolic reprogramming and MPN development (7). **(B)** Epigenetic alterations such as loss of EZH2 function in MPN may contribute to progression of disease severity *via* reprogramming of BCAA metabolism (92).

More recently, Rao et al. elegantly described a reprogramming of metabolic activity in mouse models of JAK2 mutant MPN (7). This report demonstrated that elevated expression of glycolytic enzymes in stem and progenitor cells of JAK2-mutant MPN mice correlated with enhanced glucose uptake, glycolysis and oxidative phosphorylation as well as use of the pentose phosphate pathway (7) (**Figure 1A**). In fact, enhanced erythropoiesis was so highly dependent on glucose that JAK2-mutant MPN mice became hypoglycemic with disease progression, leading to a state of “energy crisis” that likely contributed to the observed elevation of lipid catabolism. Interestingly, the survival of JAK2-exon 12 mutant MPN model mice was significantly lengthened by a high fat diet, suggesting disease could be impacted by the nutritional energy source. Rao et al. also demonstrated that PFKFB3 was elevated in cells from JAK2-mutant mice. The PFKFB3 inhibitor 3-PO induced apoptosis in MPN model cell lines and primary cells from patients, but only showed additive effects with ruxolitinib in cell lines. Similarly, therapeutic treatment of JAK2-mutant MPN mice with 3-PO modestly antagonized the MPN phenotype, and only showed modest effects in combination with ruxolitinib. Upregulation of proteins that promote enhanced glycolysis may be regulated by HIF1-α, which could provide a therapeutic target to antagonize metabolic dependencies in MPN-driving cells (93). MPN patients often gain weight during ruxolitinib treatment and Rao et al. also suggests PV patients undergoing cytoreductive therapy display increased blood sugar levels (7). Although many factors many contribute to such observations, such as effects on leptin signaling due to JAK2 inhibition or perhaps increased appetite due to reduced splenomegaly, these observations suggest a link between altered metabolic stasis and MPN (94). Ruxolitinib treatment may antagonize enhanced metabolism in MPN progenitor cells, leading to an imbalance of caloric intake and utilization, leading to weight gain (95). Targeting metabolic processes to selectively antagonize malignant progenitor cells thus could have other health concerns for patients. Nonetheless the study by Rao et al. suggests that aberrant metabolic activity of disease-driving MPN progenitor cells may provide a therapeutically targetable liability that may spare cells that aren’t dependent on aberrant metabolic regulation (7).

Numerous signaling pathways activated by JAK2 could impinge on metabolic control in MPN. Induction of PFKFB3 and glycolysis by JAK2-V617F is mediated by STAT5 activation, but other pathways likely contribute to metabolic changes induced by aberrant JAK2 signaling (87). Notably, the mTOR pathway, a well-known regulator of metabolic processes, is regulated by PI3K/AKT signaling, a downstream effector pathway of JAK2 (1, 96). mTOR inhibitors are effective in MPN models, but clinical assessment has not gained much traction, with a more recent focus on PI3K inhibitors, which target mTOR activating signals (5, 19, 23–26, 96, 97). Such signaling pathway inhibitors of course would not affect metabolism specifically. Further understanding of the deregulated metabolic processes that contribute to driving MPN is needed to determine the extent to which these could be targeted more directly, for example, with specific metabolic enzyme inhibitors.

## Epigenetic Regulator-Mediated Control of Metabolic Processes in MPN

Evidence suggests that deregulated metabolic programs are manifested by altered regulation of gene expression of key proteins in glycolytic processes as well changes of lipid and amino acid metabolism (98–100). Metabolic changes induced in MPN model mice suggest aberrant JAK2 signaling may drive these effects (7). However, other mutations or deregulated signaling associated with MPN may contribute to altered metabolic pathways in MPN driving cells. For example, recent studies have suggested altered epigenetic control of methylation and acetylation have the potential to contribute to the control of metabolic processes, such as amino acid metabolism and glycolysis in MPN.

### Methylation and Branched-Chain Amino Acid Metabolism

EZH2, as the enzymatic component of the polycomb repressive complex-2 (PRC2), catalyzes methylation of H3K27 which leads to suppression of gene expression (101). The frequency (~5–10%) of inactivating *EZH2* mutations found in MF suggests loss of EZH2-mediated methylation contributes to neoplastic disease (6, 60, 102, 103). Genetic loss of *EZH2* leads to a more advanced MPN phenotype and decreased survival in MPN mouse models, suggesting EZH2 plays a tumor suppressive role in MPNs (47, 55, 56, 92).

EZH2 loss/inactivation in an N-RAS-driven mouse model of MPN led to enhanced branched-chain amino acid metabolism (BCAA) during the progression of disease (92). This is consistent with a known role of EZH2 in the regulation of metabolic processes such as glucose, fatty acid, and amino acid metabolism in cancer cells (104). BCAA metabolism plays roles in a variety of cancer types and has been identified as a metabolic vulnerability in myeloid leukemia (105, 106). BCAA metabolism is regulated by branched chain amino acid transaminase 1 (BCAT1) in reversible reactions generating branch-chain alpha-keto acids and glutamate from BCAAs and alpha-ketoglutarate (α-KG) (105). The loss of EZH2 led to the loss of repressive methylation of the *BCAT1* promoter, leading to enhanced BCAT1 expression, sustained BCAA levels, progression of MPN (e.g., enhanced myelofibrosis) and transformation to AML (92) (**Figure 1B**). BCAT1 is generally low or not expressed in hematopoietic cells and exogenous expression of BCAT1 mimics the effects of the loss of EZH2 on leukemia initiating cells (92, 106). Importantly, leukemia initiating cells that lacked EZH2 were more sensitive to BCAT1 inhibition than normal HSPCs, which are unaffected by loss of BCAT1 (92). Thus, the loss of EZH2 can drive MPN severity and the aberrant control of post-MPN leukemic-initiating cells *via* BCAT1-mediated alteration of metabolic processes. Loss of function *EZH2* mutations in human MPN are associated with higher levels of BCAT1 mRNA compared to patients with wildtype *EZH2* (92). Therefore, the loss of EZH2-mediated inhibition of BCAT1 expression may contribute to altered metabolic profiles in MPN-driving cells, which may explain the poor prognosis of MPN patients who harbor *EZH2* mutations (60).

The increase in BCAT1 levels associated with inactivating mutations of *EZH2* could contribute to the available pool of α-KG, an important component of the TCA cycle that also functions as a co-factor for the TET2 methylcytosine dioxygenase that promotes DNA demethylation (107, 108). Loss of function mutations in *TET2* are present in 10% to 20% of MPN patients where they contribute to clonal dominance and disease initiation and associate with poor outcomes (6, 61, 109, 110). Loss of TET2 leads to distinct gene expression profiles and enhances disease in an MPN mouse model (53). Likewise, IDH1/2 mutations lead to the production of 2-hydroxyglutarate instead of α-KG, which effectively inhibits the function of TET2, and thus such mutations mimic loss of function of TET2 (111, 112). This is a clear example of the interconnectivity between alterations in metabolic pathways leading to deregulation of epigenetic regulatory mechanisms in cancer. Mutations of *IDH1/2* are present in 1% to 3% of MPN patients, are enriched in post-MPN leukemia (~20%) where they associate with poor survival, and enhance the progression of disease in MPN model mice (48, 62). In the MPN model driven by activated N-RAS and loss of *EZH2*, with subsequent elevation of BCAT1, changes in cellular α-KG levels were not detected (92). However, BCAT1 expression in non *IDH* or *TET2* mutant AML stem cells correlates with decreased α-KG levels and gene expression profiles similar to *IDH* mutant cells, suggesting deregulation of methylation regulated by TET2 (113). Whether BCAT1 levels affect the activity of TET2-mediated regulation of DNA methylation and gene expression in MPN patients is unknown.

Loss of EZH2 function has been proposed to play a role in the effects elicited by other mutations found in MPN, including *ASXL1* and *SRSF2*, suggesting EZH2 loss of function may contribute to the biology of disease in patients beyond those that have *EZH2* mutations (57, 114). As normal HSPCs do not require BCAT1 and these cells have low BCAT1 expression, BCAT1 levels may define subsets of MPN patients that could exhibit BCAT1-driven therapeutic liabilities. BCAT1 inhibition could provide a therapeutic strategy for MPN patients who have elevated BCAT1 (e.g., patients with *EZH2*, and possibly *ASXL1* and *SRSF2* mutations) to shift malignant myeloproliferative hematopoiesis back to normal hematopoiesis.

Moreover, the associated decrease in H3K27 methylation with EZH2 loss of function leads to an increase in H3K27 acetylation, providing binding sites for BRD4 and enhancement of gene expression. This epigenetic switch reverts a transcriptional inhibitory mark (methylation) to an activation mark (acetylation), effectively creating a synthetic lethal interaction between loss of function *EZH2* mutations and BET inhibition in mouse models (47). As such, loss of EZH2 function in MPN patients may provide enhanced sensitivity to BET inhibitors, which are currently in clinical testing for myelofibrosis patients. It will be interesting to see the relative bromodomain inhibitor responses of MPN or post-MPN AML patients who have inactivating *EZH2* mutations, or *ASXL1* or *SRSF2* mutations which antagonize PRC2/EZH2 function (57, 114).

Finally, BCAA metabolism has recently been associated with tyrosine kinase inhibitor resistance in lung cancer, suggesting such metabolic reprogramming may also contribute to the inefficacy or resistance to JAK2 inhibition (115). In fact, MPN patients with multiple mutations, including in *EZH2* and *ASXL1*, in addition to a JAK2 activating mutation tend to respond poorly to JAK2 inhibitor therapy (59). The possibility exists that BCAA metabolism may affect response to JAK2 inhibition or may contribute to an adaptive response in cells exposed to chronic JAK2 inhibition. If so, inhibition of BCAA metabolism may enhance the efficacy of current targeted therapies in subsets of MPN patients.

### Metabolic Control by Histone Deacetylases

Protein lysine acetylation is not limited to histones as it is a key modification found throughout the human proteome, affecting most major cellular processes including metabolism (116–118). One study indicates that almost every enzyme within major metabolic pathways is acetylated, clearly suggesting a likely role of acetylation in metabolic control (118). For example, phosphoenolpyruvate carboxykinase is a gluconeogenic enzyme whose protein stability and levels are antagonized by acetylation (118). Given the breadth of the acetylated proteome and the non-selectivity of clinically assessed HDAC inhibitors, it is likely specific functions of individual HDAC family members need to be identified, along with the development of HDAC-isoform specific inhibitors, in order to selectively target HDACs to impede specific regulatory mechanisms of acetylation. While pan-HDAC inhibitors (e.g., vorinostat, givinostat, pracinostat, panobinostat) have been assessed in clinical trials and some have displayed efficacy in MPN patients, toxicity concerns may limit their potential (119–126). This suggests the development of more specific HDAC inhibitors may minimize adverse effects and allow this class of epigenetic regulators to be therapeutically targeted.

Recent reports have also highlighted a significant role for HDAC11, which has roles in immune tolerance, in regulating metabolic processes (127–130). We, along with our collaborators, demonstrated a role for HDAC11 in HSPCs in a transplantation mouse model of MPN driven by MPL-W515L. Data obtained using *HDAC11*-null mice and MPN patient samples treated with selective HDAC11 inhibitors suggest that HDAC11 contributes to the neoplastic nature of MPN cells but not normal hematopoiesis (50). Subsequent studies using global acetylomic profiling following HDAC11 inhibition identified glycolytic enzymes (e.g., enolase-1 (ENO-1)) as potential substrates of HDAC11. Pharmacological inhibition as well as knockdown of HDAC11 increased the acetylation of ENO-1, decreased the activity of ENO-1, and reduced the rate of glycolysis as well as oxidative phosphorylation in MPN model cell lines and primary cells from MPN patients but not healthy controls (131). These observations are supported by previous studies linking acetylation to metabolic processes and potentially provide HDAC11-specific functions in regulating metabolic pathways (118).

Therefore, HDAC11 may contribute to the MPN-driver associated metabolic changes that contribute to MPN pathogenesis (7). However, specific metabolic effects of HDAC11 depletion in MPN mouse models have yet to be determined. Similarly, the efficacy of a selective anti-HDAC11 therapeutic in such models and the effects of such a therapeutic on the metabolic profiles of MPN-driving cells are unknown. Continued development of such inhibitors are required to ascertain the extent to which HDAC11 inhibition may provide a therapeutic option to disrupt control of cellular processes requisite for MPN-driving HSPCs, and to determine if the observed metabolic vulnerabilities that have been identified in MPN-driving HSPCs are regulated by HDAC11 (7). HDAC11 also displays a recently identified deacylase activity and the role of this activity in MPN formation is unknown (132–134). Interestingly, HDAC11 was the most highly induced HDAC in response to a variety of HDAC inhibitors in AML cells, suggesting it indeed may have unique and critical properties compared to other HDAC family members (135). HDAC11 inhibition has been suggested to overcome therapy resistance in lung cancer models, and while its role in resistance to targeted therapies such as approved JAK2 inhibitors in MPN is unknown, current data suggest potential for the combination of JAK2 inhibition and HDAC11 inhibition as a possible future therapeutic strategy (136).

## Discussion

MPN-driver mutations enhance JAK2 signaling which promotes neoplastic HSPC expansion, and altered epigenetic control mechanisms play an etiologic role in the development and progression of MPN. Recent studies provide evidence that altered metabolic control may play an important role in MPN and that altered epigenetic regulation may contribute to neoplastic metabolic profiles. Deregulated metabolic processes that support and drive MPN phenotypes may reveal novel vulnerabilities that can be targeted to suppress the malignant clone while sparing healthy HSPCs. The role of disease-associated metabolic states in the upfront inefficacy of JAK2 inhibitors, as well as whether or not JAK2 inhibitor therapy induces changes in metabolic states that contribute to JAK2 inhibitor failure, are questions that remain important to address. Further understanding the metabolic profiles of MPN HSPC clones in MPN subtypes, genotypes, responses to JAK2 inhibitor therapy, and disease progression are required in order to understand the potential relationship between deregulated epigenetics and metabolism in MPN, which could lead to the development of much needed remission-inducing personalized therapies for patients.

## Author Contributions

All authors contributed to the article and approved the submitted version.

## Conflict of Interest

GR receives research funding from Incyte Corporation and Revolution Medicines, Inc. for projects not related to this manuscript. PE-B received research funding from Incyte Corporation and Forma Therapeutics. PE-B is the Chief Scientific Officer of Ibis Therapeutics and works on drug development unrelated to this manuscript. KW received research funding from Forma Therapeutics and Tolero Pharmaceuticals.

The remaining author declares that the research was conducted in the absence of any commercial or financial relationships that could be construed as a potential conflict of interest.
